# Supplementary material for: An Example of Personalized Treatment in HR+ HER2+ Long Survivor Breast Cancer Patient (Case Report)
Source: Curr Oncol. 2021 May 25;28(3):1980–7. doi: 10.3390/curroncol28030184 (PMC8161821; doi:10.3390/curroncol28030184)
Supplement: Supplementary file 1 [file curroncol-28-00184-s001.zip › curroncol-1167217-SI.pdf]

Supplementary Materials:

An Example of Personalized Treatment in HR+ HER2+ Long Survivor Breast Cancer Patient (Case Report)

Martina Panebianco, Beatrice Taurelli Salimbeni, Michela Roberto and Paolo Marchetti

Table S1. Panel of SNPs investigated.

Gene	Rs Code	Polymorphism	Amino acid	Genotype	Functional effects
			TRANSPORTERS		
**ABCB1**	**rs1128503**<br>**rs1045642**<br><br>**rs4148738**	**C1236T**<br>**C3435T**<br><br>**G>A**		**C/T**<br>**C/T**<br>**T allele leads to lower activity**<br>**G/A G allele increased Dabigatran**	**Decreased**
**ABCC1/MRP1**	rs45511401	G761V-G>T		G/G normal activity	Normal activity
**ABCC2/MRP2**	rs8187710		Cys1515Tyr	G/G normal activity	Normal activity
**ABCC2/MRP2**	rs17222723	T > A	Val1188Glu	T/T normal activity	Normal activity
**ABCC2-C-24T**	**rs717620**			**T/T**<br>**T allele leads to lower expression**	**Lower Expression-**
**ABCG2**	rs2231142	C421A	Q141K	C/C	
**SLC15A2**	rs2257212	C > T	L305F	C/C	
**SLCO1B1**	**rs4363657**<br>**rs4149056**	**T521C**<br>**T>C**	**V174A**	**T/C**<br>**T/C**<br>**C allele leads to lower activity of transporter**	**Decreased activity**
**5HTT-LPR**				**S/L**<br>**S allele leads to lower gene transcription**	**Decreased activity**
			CYP		
**CYP1A2*1C**	rs2069514	G3860A		G/G normal activity	Normal activity
**CYP1A2*1F**	**rs762551**	**C163A**	**Intron**	**A/A A allele leads to increased inducibility**	**Increased**
			CYP2C19<br>Normal activity		
**CYP2C19*17**	rs12248560	C806T	Upstream	C/C	Normal activity
**CYP2C19*10**	rs6413438	C1953T		C/C	
**CYP2C19*2**	rs4244285	G19154A		G/G	
**CYP2C19*3**	rs4986893	G17948A		G/G	
**CYP2C19*4**	rs28399504	A1G		A/A	
**CYP2C19*5**	rs56337013	C90033T		C/C	
**CYP2C19*7**	rs72558186	T19294A		T/T	
**CYP2C9*2**	rs1799853	C430T	Cys144Arg	C/C	
**CYP2C9*3**	**rs1057910**	**A1075C**	**Ile359Leu**	**A/C**<br>**C allele leads to lower activity**	**Decreased**
			**CYP2D6**<br>**Poor metabolizer**		

**CYP2D6*2**	rs16947	C2850T	Arg296Cys	C/C	
**CYP2D6*10**	**rs1065852**	**C100T**	**Pro34Ser**	**T/T**	
**CYP2D6*17**	rs28371706	C1023T	Thr107Ile	C/C	
**CYP2D6*6**	rs5030655	1707delT	Trp152fs	IN3/IN3	
**CYP2D6*20**	rs72549354	insG	Leu213fs	DEL/DEL	
**CYP2D6*5**	-	Deletion	Stop codon	Absent	
**CYP2D6*38**	rs72549351	delGACT	Thr272fs	INS/INS	
**CYP2D6*29**	rs61736512	G1659A	Val136Met	G/G	
**CYP2D6*2A**	rs1080985	C1584G	Upstream	C/C	
**CYP2D6*3**	rs35742686	2549delA	Arg259fs	INS/INS	
**CYP2D6*4**	**rs3892097**	**G1846A**	**Acceptor**	**A/A**	
**CYP2D6*41**	rs28371725	G2988A	Intron	G/G	
**CYP2D6*7**	rs5030867	A2935C	His324Pro	A/A	
**CYP2D6*9**	rs5030656	2615_2617delAAG	Inframe del.	INS/INS	
**CYP3A4*1B**	rs2740574	A392G	Upstream	A/A	Normal activity
**CYP3A4*22**	rs35599367	C15389T	Intron	C/C<br>G alleles lead to in-creased activity	Normal activity
**CYP3A5*3**	rs776746	A6986G	Acceptor	G/G	Normal activity
**CYP1A1**	rs1048943	T>C	Ile462Val	T/T<br>C allele leads to in-creased activity	Normal activity
**CYP2B6**	**rs2279343**	**A785G**	**Lys262Arg**	**A/G**	**Decreased (lower activity)**
	**rs3745274**	**G516T**	**Gln172His**	**G/T**	
	**rs3211371**	**C1459T**	**Arg487Cys**	**C/T**	
	**rs2279343**	**A785G**	**Lys262Arg**	**A/G**	
**CYP2B6**	rs28399499	T983C	Ile328Thr	T/T	Normal activity
**CYP2A6**	rs28399433<br>rs1801272	A48C<br>T>A		A/A<br>T/T	Normal activity
**CYP2C8*2**	rs11572103	A805T	Ile269Phe	A/A	Normal activity
**CYP2C8*4**	rs1058930	C792G	Ile264Met	C/C	Normal activity
**OTHER DRUG METABOLIZING ENZYMES**					
**TPMT*2**	rs1800462	C>G	Ala80Pro	C/C	Normal activity
**TPMT*3B**<br>**TPMT*3C**	rs1800460<br>rs1142345	G460A<br>A719G	Ala154Thr<br>Tyr240Cys	G/G<br>A/A	Normal activity
**UGT1A1*28**	rs8175347	(TA)(n) repeat sequence	(6/7TA) TATA box	6/6	Normal activity
**UGT2B17**		Delection		Norma Genotype	Normal activity
**DPYD**	rs2297595	A166G		A/A	Normal activity
	rs67376798	A2846T	Asp949Val	A/A	
**DPYD*6**	rs1801160	G>A	Val732Ile	G/G	Normal activity
**DPYD*2A**	rs3918290	IVS 14+1G>A		G/G	-
**COMT**	**rs4680**<br>**rs4633**	**G>A**<br>**C>T**	**Val158Met**<br>**His62His**	**G/A**<br>**C/T**	**Decreased**
**COMT**	**rs4818**	**C>G**	**Leu136Leu**	**C/G**<br>**G alleles lead to in-creased activity**	**Increased**
**MTHFR**	rs1801133	C677T	Ala222Val	T/T	Normal activity
**MTHFR**	**rs1801131**	**A1298C**	**Glu429Ala**	**A/A**	**Decreased**
**TSER-**	**rs45445694**	**28bpVNTR**		**3R/3R**	**Increased transcription**
**CBR3**	rs1056892	G>A	Val244Met	G/G	Normal activity
**CYBA**	rs4673	C242T		C/T	
**NCF4**	rs1883112	A212G		G/G	
**RAC2**	rs13058338	T7508A		T/A	
**NAT1*14B**	rs4986782	G560A	Arg187Gln	G/G	Normal activity
**NAT1*15B**	rs5030839	C599T	Stop Codon	C/C	Normal activity
**NAT1*17B**	rs56379106	C190T	Arg64Trp	C/C	Normal activity

**NAT1*22B**	rs56172717	A752T	Asp251Val	A/A	Normal activity
**NAT2*3**	**rs1801280**	**T341C**	**Ile114Thr**	**C/C<br>C allele lead to lower activity**	**Decreased**
**NAT2*6<br>NAT2*7**	rs1799930<br>rs1799931	G590A<br>G857A	Arg197Gln<br>Gly286Glu	G/G<br>G/G	Normal activity
**UMPS**	rs1801019	G213A<br>G>C	Gly213Asp<br>Gly213Ala	G/C	-
**CBS**	rs234706	C699T		C/T<br>Allele T associated with increased risk of ammonia and sulphite detoxification defects	
**MTR**	rs1805087	A2756G		A/G	Increased cardiovascular risk
**MTRR**	rs1801394	A66G		A/G	Increased cardiovascular risk
**RECEPTORS**					
**DRD2/ANKK1-Q713K**	rs1800497	C957T		C/C	Normal activity
**DRD2-141-insC/delC**	rs1799732	insC/delC	Upstream	InsC/InsC	Normal activity
**DRD2-S311C**	rs1801028	C932G	Ser311Cys	C/C	Normal activity
**DRD3-S9G**	rs6280	A25G	Gly9Ser	A/A	Normal activity
**HTR2A**	rs6314<br>rs7997012<br>rs6311	C1354T<br>A>G<br>G1438A	His452Tyr<br>Intron<br>Upstream	C/T<br>A/G<br>A/A	
**HTR2C**	rs6318	G68C	Cys23Ser	G/G	
**OPRM1**	rs1799971	A118G	Asn40Asp	A/G	More pain
**VDR-Bmsl**	rs1544410	C>T		C/C	
**VDR-Taql**	rs731236	A>G		A/A	
**VDR-Apa**	rs7975232	C>A		C/A	
**VDR**	rs4516035	1012T>C		T/T	
**AGT**	rs4762	C9369T	T174M	C/T<br>T allele leads to high level of angiotensinogen and increased cardiovascular risk	Increased cardiovascular risk
**AGT**	rs699	T9543C	M235T	C/C<br>C allele leads to increased cardiovascular risk and sodium related hypertension	Increased cardiovascular risk
**AGTR1**	rs5186	A1166C		A/C<br>C allele leads to hypertension	Increased cardiovascular risk
**ITGB3**	rs5918	T1565C		T/T	Normal activity
**OXIDATION REDUCTION ENZYMES**					
**GSTP1**	**rs1695<br>rs1138272**	**A313G<br>C>T**	**Ile105Val<br>V114A**	**A/G<br>C/C<br>G alleles lead to lower activity**	**Decreased**
**GSTM1**		**Deletion**		**Deleted**	**No activity**
**GSTT1**		Deletion		Normal	Normal activity
**DNA REPAIR ENZYMES**					
**ERCC1**	rs3212986	C8092A	3 Prime UTR	C/A	-

	rs11615	T19007C	Asn118=	T/C	
**XRCC1**	rs25487	G28152A	Gln399Arg	G/A	
			**LIPID METABOLISM**		
**APOA1**	rs1799837	G75A		G/G<br>*Protective against cardiovascular event*	
**APOB1**	rs5742904	G10580A	Arg3500Gln	G/G	
**APOE**	rs7412<br>rs429358			E3/E3	Normal activity
**CETP**	rs1532624	C>A	Intron	C/C	Normal activity
**HMGCR**	rs3761740<br>rs5908	C911A<br>A>G	Upstream tra.<br>Ile585Val	C/C<br>A/A	Normal activity<br>Normal activity
**PON1**	rs662<br>rs705379	A575G<br>C108T	Gln192Arg<br>Upstream	A/A<br>C/C	Normal activity
			**COAGULATION ENZYMES**		
**VKORC**	rs9923231	G1639A	Upstream	G/A	Decreased
	rs9934438	C1173T	Intron	C/T<br>A/T alleles lead to less coagulating capacity	
**CES1**	rs2244613<br>rs8192935	A>C<br>C>T	Intron	A/A<br>C/C	Normal activity
**F II**	rs1799963	G20210A	3 Prime UTR	G/G	Normal activity
**PAI**	rs1799889	4G/5G	Upstream	4G/4	
**F V**	rs6025	G1691A	Arg506Gln	G/G	
**β-fibrinogen**	rs1800790	G455A	-	G/A<br>A allele	Increased level of fibrinogenous
**FattoreXIII**	rs5985	G>T	Val135Leu	G/T	

Abbreviation: ABCB1, ATP binding cassette subfamily B member 1; ABCC1, ATP Binding Cassette Subfamily C Member 1; MRP1, Multidrug resistance-associated protein 1; ABCC2, ATP Binding Cassette Subfamily C Member 2; MRP2, Multidrug resistance-associated protein 2; ABCG2, ATP-binding cassette sub-family G membro 2; SLC15A2, Solute Carrier Family 15 Member 2; SLCO1B1, solute carrier organic anion transporter family member 1B1; 5-HTTLPR, serotonin-transporter-linked polymorphic region; CYP1A2, Cytochrome P450 Family 1 Subfamily A Member 2; CYP2C19, cytochrome P450 family 2 subfamily C member 19; CYP2D6 Cytochrome P450 family 2 subfamily D member 6; CYP3A4, Cytochrome P450 family 3 subfamily A member 4; CYP3A5, Cytochrome P450 family 3 subfamily A member 5; CYP1A1 Cytochrome P450 family 1 subfamily A member 1; CYP2B6 Cytochrome P450 family 2 subfamily B member 6; CYP2C8, Cytochrome P450 family 2 subfamily C member 8; TPMT, Thiopurine methyltransferase; UGT1A1, uridine-diphosphoglucuronate glucuronosyltransferase; DPYD, dihydropyrmidinase; COMT, catechol O-methyltransferase; MTR, methionine synthase; TSER, synthase enhancer region; CBR3, carbonyl reductase 3; MTHFR, methylenetetrahydrofolate reductase; TYMS, thymidylate synthase; NAT1, Arylamine N-acetyltransferase 1; NAT2, Arylamine N-acetyltransferase 2; UMPS, Uridine Monophosphate Synthetase; DRD2, dopamine receptor D2; ANKK1, Ankyrin repeat and kinase domain containing 1; HTR2A, 5-Hydroxytryptamine Receptor 2A; HTR2C, 5-Hydroxytryptamine Receptor 2C; OPRM1, Opioid Receptor Mu 1; VDR, vitamin D receptor; AGT, angiotensinogen gene; ITGB3, integrin subunit beta 3; GSTP1, Glutathione S-Transferase P1; GSTM1, glutathione S-transferase Mu 1; GSTT1, Glutathione S-Transferase Theta 1; ERCC1,Excision Repair 1, Endonuclease Non-Catalytic Subunit; XRCC1, X-ray repair cross complementing protein 1; APOA1, Apolipoprotein A1; APOB1, Apolipoprotein B1; CETP, Cholesteryl ester transfer protein; HMGCR, 3-Hydroxy-3-Methylglutaryl-CoA Reductase; PON1, paraoxonase/arylesterase 1; VKORC, Vitamin K epOxide Reductase Complex; CES1 Carboxylic ester hydrolase; F II, coagulation factor II; PAI, Plasminogen Activator Inhibitor; F V, coagulation factor V; CBS, cystathionine beta-synthase; F XIII, coagulation factor XIII;

Colour legend

Increased activity

Decreased activity

Absent activity
